# The Sigma-1 Receptor in Cellular Stress Signaling

**DOI:** 10.3389/fnins.2019.00733

**Published:** 2019-07-16

**Authors:** Teruo Hayashi

**Affiliations:** Seiwakai Nishikawa Hospital, Hamada, Japan

**Keywords:** sigma-1 receptor, endoplasmic reticulum, ER stress, Ca^2+^, oxidative stress

## Abstract

After decades of research, the sigma-1 receptor (Sig-1R)’s structure, and molecular functions are being unveiled. Sig-1R is an integral endoplasmic reticulum (ER) membrane protein which forms an oligomer and binds a variety of psychotropic drugs. It forms a complex with the ER chaperone BiP that controls specific signaling molecules’ stability and function at the ER to regulate Ca^2+^ signaling, bioenergetics, and ER stress. Sig-1R is highly enriched in ER subdomains that are physically linked to outer mitochondrial membranes, reflecting its role in regulating ER–mitochondria communications. Thus, Sig-1R ligands are expected to serve as novel neuroprotective agents which treat certain psychiatric and neurodegenerative disorders. In this short review, the cell biological aspects of Sig-1R are discussed, with a particular focus on its role in fundamental ER functions.

## Introduction

The sigma receptor was originally proposed to be a subtype of the G protein-coupled opioid receptors (Su and Hayashi, 2003). However, a series of later experiments confirmed that the sigma receptor is insensitive to naloxone and GTP (Wilke et al., 1999; Su and Hayashi, 2003). Although the sigma-1 receptor (Sig-1R), a subtype of the sigma receptor, has largely been a mystery since its existence was first proposed in the 1970s, its ligands have demonstrated therapeutic potential in a variety of situations (Su and Hayashi, 2003). At the cellular level, the major action site of Sig-1R is the ER (Su et al., 2010). However, it was recently shown that it is highly enriched in a specialized subcomponent of the ER membrane called the mitochondrial-associated ER membrane (MAM) (Hayashi and Su, 2007). Sig-1R ligands modulate cellular functions, such as ion channel activity, neuronal firing, neuronal differentiation, cancer growth, and cell death/apoptosis, as well as behaviors related to neurological and neuropsychiatric disorders such as substance addiction (e.g., cocaine and methamphetamine abuse), depression, schizophrenia, amyotrophic lateral sclerosis, and Alzheimer’s disease (Mavlyutov et al., 2010, 2013; Su et al., 2010; Maurice and Goguadze, 2017; Nguyen et al., 2017). Uniqueness of Sig-1R ligands lies in their mode of action; in experimental animals, they only exert a therapeutic effect under pathological conditions, particularly ER or mitochondrial stress, and have no effect in normal naïve animals (Hayashi and Su, 2004, 2007; Maurice and Goguadze, 2017; Nguyen et al., 2017). In general, they act to normalize physiological or behavioral functions (Hayashi and Su, 2004). The unique “normalizing” effect of Sig-1R ligands may be partially attributed to Sig-1R’s characteristics as a stress protein and molecular chaperone. In this short review, Sig-1R’s cell biological aspects are discussed, with a particular focus on its role in fundamental ER functions.

## Structure of Sig-1R

Recent studies have ascertained that Sig-1R forms oligomers (e.g., trimers) at the ER membrane, creating a Sig-1R ligand–binding pocket (Chu et al., 2013; Schmidt et al., 2016). A crystallographic study demonstrated that Sig-1R has a single transmembrane domain in the middle of the protein, although other studies have proposed models with two transmembrane domains (Ortega-Roldan et al., 2015; Schmidt et al., 2016; Penke et al., 2018). Although the crystallographic study proposed the C-terminus localizing at the cytoplasmic milieu, a recent study using biologically, and physiologically relevant membranes clearly demonstrated that the Sig-1R possesses a long ER-lumenal domain including the C-terminus (Schmidt et al., 2016; Mavlyutov et al., 2017). By expressing ascorbate peroxidase 2 (i.e., APEX2)-tagged Sig-1R in ND7/23 cells or in dorsal root ganglion neurons, Mavlyutov et al. (2017) demonstrated that (i) N-terminus of the Sig-1R faces the cytosol while the C-terminus faces the ER lumen; (ii) the transmembrane domain exists between amino acids 1–80. The C-terminal hydrophobic domain seems to be involved in forming oligomers (Schmidt et al., 2016). Size exclusion chromatography showed that Sig-1R ligands mediate alterations in Sig-1R oligomerization (Gromek et al., 2014). Meanwhile, FRET studies further support the previous finding that Sig-1R agonists and antagonists tend to cause oligomer dissociation and stabilization, respectively (Mishra et al., 2015; Yano et al., 2018).

## Role of the Sig-1R–BiP Protein Complex in Sig-1R Activation

An assay screening Sig-1R binding proteins identified BiP, an ER molecular chaperone, as one of the major components of the Sig-1R protein complex (Hayashi and Su, 2007). Solution NMR spectroscopy showed that the C-terminal membrane–tethering domain of Sig-1R interacts with full-length BiP or the nucleotide-binding domain of BiP (Ortega-Roldan et al., 2013). This finding raised the unexpected possibility that Sig-1R regulates chaperone activity at the ER. The interaction between Sig-1R and BiP does not change either protein’s stability, excluding the possibility that Sig-1R is a substrate of the BiP chaperone (Hayashi and Su, 2007). A light-scattering assay provided clear evidence that the purified C-terminus of the Sig-1R polypeptide prevents heat-induced protein aggregation *in vitro* (Hayashi and Su, 2007), suggesting that Sig-1R possesses an innate biological activity similar to that of molecular chaperones. Interaction between purified BiP and the C-terminus of Sig-1R blocks this activity (Hayashi and Su, 2007). Immunoprecipitation and BRET assays have further examined the physiological role of the heteromeric interaction between Sig-1R and BiP. They revealed that (+)-pentazocine causes Sig-1R and BiP to dissociate, but haloperidol inhibits this dissociation (Hayashi and Su, 2007; Yano et al., 2018). As mentioned above, Sig-1R agonists, such as (+)-pentazocine, induce the dissociation of Sig-1R oligomers, but Sig-1R antagonists, such as haloperidol, stabilize them (Gromek et al., 2014; Mishra et al., 2015; Yano et al., 2018). It is likely that Sig-1R multimerization, which is regulated by Sig-1R ligands, is a key factor promoting Sig-R–BiP interaction.

Taken together, the research results to date clearly indicate Sig-1R is a unique chaperone protein that forms homo-oligomers, and interacts with BiP in its dormant state. Upon binding Sig-1R agonists, which mediate the homo-oligomers’ destabilization, and thus lead to dissociation of BiP from Sig-1R, Sig-1R is activated to exert its innate chaperone activity.

## Roles of Sig-1R in Regulating ER Functions

**FIGURE 1:**
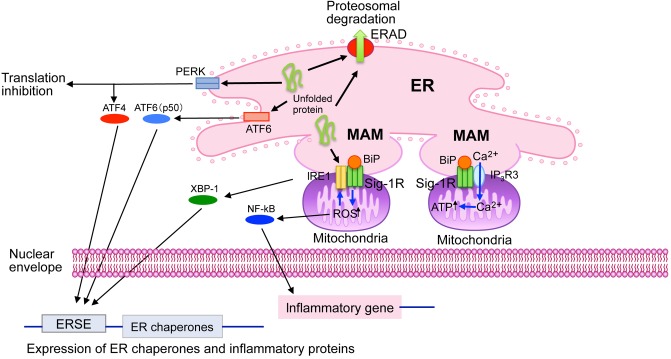
Cell biological roles of sigma-1 receptors (Sig-1R) at the ER. Sig-1R are highly enriched at the mitochondria-associated ER membrane (MAM). Sig-1R is a unique chaperone protein that forms homo-oligomers (i.e., trimmers) and interacts with BiP in its dormant state. Upon binding Sig-1R agonists, which mediate the homo-oligomers’ destabilization, and lead to dissociation of BiP from Sig-1R, Sig-1R is activated to exert its innate chaperone activity. Sig-1R stabilizes IP3 receptors type-3 (IP_3_R3) at the MAM, thus regulates Ca^2+^ influx into mitochondria and ATP synthesis. Sig-1R also stabilizes the ER stress sensor IRE1 at the MAM under ER or oxidative stress. Sig-1R thus regulates expression of stress-related proteins by regulating transcription factors XBP-1 and NF-kB. ERAD, ER-associated degradation; ERSE, endoplasmic reticulum stress element.

The endoplasmic reticulum is a multifunctional intracellular organelle that serves as an intracellular Ca^2+^ store and a factory of protein and lipid synthesis (Hayashi et al., 2009; Penke et al., 2018). Recent discoveries highlight that the ER also regulates stress response (namely the ER stress response) by transmitting signals derived from unfolded proteins accumulating in the ER (Mori, 2015; Penke et al., 2018). The ER dynamically changes its shape by forming a reticular structure throughout the entire cytoplasm, thus enabling physical connections to other subcellular organelles, such as the plasma membrane and mitochondria (Hayashi et al., 2009; Kerkhofs et al., 2017). As discussed below, Sig-1R appears to be involved in these fundamental ER functions (Figure 1).

### Ca^2+^ Signaling and Bioenergetics

Sig-1R is enriched at ER membranes that are physically associated with the mitochondria [i.e., mitochondria-associated membranes (MAMs)] (Hayashi et al., 2009; Penke et al., 2018). The physical membrane contact between the ER and the mitochondria enables the ER to directly provide Ca^2+^ to mitochondria via IP3 receptors at MAMs, thus regulating bioenergetics, and free radical formation in mitochondria (Hayashi, 2015b). This Ca^2+^, provided via MAMs, activates enzymes involved in the TCA cycle, thus potentiating ATP production (Hayashi et al., 2009; Hayashi, 2015b). Sig-1R at MAMs stabilizes the activated IP3 receptor type 3, which is abundant at MAMs, thus ensuring proper Ca^2+^ influx into the mitochondria from the ER (Hayashi and Su, 2007) (Figure 1).

### ER Stress, Oxidative Stress, and Cellular Survival

Endoplasmic reticulum stress sensor proteins monitor the concentration of misfolded proteins in the ER lumen and regulate molecular chaperones’ expression (Mori, 2015). The stress sensor protein IRE-1 is enriched at MAMs although other ER stress sensor proteins, such as PERK and ATF-6, are not (Mori et al., 2013). Sig-1R stabilizes IRE-1 at the MAM (Figure 1). Sig-1R associates with IRE-1 only when IRE-1 is activated (i.e., phosphorylated) under ER stress, prolonging IRE-1’s innate endonuclease activity in CHO cells (Mori et al., 2013). Activated IRE-1 promotes XBP1 mRNA splicing to express the XBP1 transcription factor, which induces the upregulation of several ER chaperones (Mori, 2015). In neonatal cardiomyocytes, Sig-1R knockdown decreases activated IRE-1 and XBP1 as well as XBP1’s nuclear localization but also increases the expression of several ER stress-related proteins, such as CHOP (Alam et al., 2017).

Sig-1R exerts a neuroprotective effect in the brain by suppressing ER stress. Sig-1R agonists improve behavioral recovery and reduce infarction sizes in mice subjected to transient cerebral artery occlusion. Importantly, treatment with Sig-1R ligands induces Sig-1R upregulation and reduces ER stress in these same mice (Morihara et al., 2018). In contrast, in Sig-1R knockout mice, activation of ER sensor proteins and downregulation of anti-apoptotic Bcl2, induced by occlusion–reperfusion of the carotid artery, was potentiated (Zhao et al., 2019). In iPS cell–originated human cortical neurons, the Sig-1R ligand N,N-dimethyltryptamine (DMT), an endogenous Sig-1R ligand (Fontanilla et al., 2009), promotes cellular survival under hypoxia in an HIF1-independent manner (Szabo et al., 2016). Knockout of Sig-1R increases α-synuclein (αSyn) oligomers, fibrillar αSyn, and αSyn phosphorylation in the dopaminergic neurons of mouse brains. However, αSyn phosphorylation is suppressed by salubrinal, an ER stress inhibitor (Hong et al., 2017). Vanishing white matter (VWM) disease is caused by eIF2B gene mutations that render the neurons prone to ER stress; primary astrocytes isolated from VWM mutant mice exhibit hypersensitivity to ER stress (Atzmon et al., 2018). Sig-1R agonists increased the mutant astrocytes’ cellular survival under ER stress, but this phenomenon was not observed in wild-type astrocytes (Atzmon et al., 2018). Thus, Sig-1R acts as a suppressor of ER stress-induced neuronal damage both *in vitro* and *in vivo*.

It seems that Sig-1R also regulates generation of reactive oxygen species (ROS) in the mitochondria (Mori et al., 2013; Hayashi, 2015a). The accumulation of mitochondrial ROS triggers the association of Sig-1R with IRE-1 to stabilize the latter (Mori et al., 2013). Sig-1R knockdown induces ROS accumulation as well as NFkB activation, whereas Sig-1R overexpression inhibits ROS generation in CHO cells (Meunier and Hayashi, 2010). In cancer cells, Sig-1R knockdown inhibits cellular proliferation by inducing ER stress as well as ROS generation (Happy et al., 2015). Two-dimensional gel electrophoresis and mass spectrometry screening of the wild type and Sig-1R knockout livers found significant changes in protein levels of the antioxidant protein peroxiredoxin 6 and the BiP (Pal et al., 2012). More importantly, the same study demonstrated that Sig-1R promotes activation of the antioxidant response element (ARE) to upregulate NAD(P)H quinone oxidoreductase 1 and superoxide dismutase 1 mRNA expression (Pal et al., 2012).

### Protein Synthesis and Trafficking

An *in vitro* study demonstrated that Sig-1R agonists potentiate BDNF secretion without affecting BDNF polypeptide synthesis (Fujimoto et al., 2012). Sig-1R seems to enhance BDNF secretion by promoting BDNF post-translational processing (Fujimoto et al., 2012). Several studies have demonstrated that Sig-1R modulates the activity of ion channels, such as Kv2.1 and Kv1.2, via physical contact (Fontanilla et al., 2009; Mavlyutov et al., 2010; Kourrich et al., 2013). Though it has been convincingly demonstrated that Sig-1R is enriched at the MAM (Hayashi and Su, 2007), it is also highly enriched at ER membranes physically linked to postsynaptic plasma membranes in specific types of neurons (e.g., those at the spinal cord) (Mavlyutov et al., 2010). Interestingly, Sig-1R has been shown to co-localize with Kv2.1 potassium channels on specialized ER membranes in spinal cord neurons (Mavlyutov et al., 2010, 2013). These findings raise the intriguing possibility that Sig-1R regulates potassium channel activity by regulating trafficking Kv2.1 channels between the ER and plasma membrane (Hayashi, 2015b). It has also been shown that the Kv2.1 potassium channel plays a role in tethering the ER to the plasma membrane (Johnson et al., 2018). Whether Sig-1R is involved in the tethering action of Kv2.1 is unclear at present. An atomic force microscopic study demonstrated that the Sig-1R directly bind to the voltage-gated ion channel (Balasuriya et al., 2012), possibly via the interaction between transmembrane domains of the channel, and the cholesterol-binding domain of the Sig-1R. Since these two molecules mostly colocalize at the cytoplasmic region of the cell, the major site of the protein interaction might involve the ER membrane (Balasuriya et al., 2012).

### Lipid Synthesis

The mitochondria-associated ER membranes is a key structure for ER lipid synthesis (Hayashi et al., 2009). For example, phosphatidylserine is synthesized at the MAM before transportation to the mitochondria for conversion to phosphatidylethanolamine (Hayashi et al., 2009). Similarly, the synthesis of steroids and sphingolipids also relies on the physical contact between the ER MAM and mitochondria (Hayashi et al., 2009). Accordingly, the MAM contains considerable levels of ceramides and cholesterol, in contrast to the other ER membranes (Hayashi and Fujimoto, 2010). Intriguingly, the Sig-1R is shown to bind specific types of sterols and sphingolipids (Ramachandran et al., 2009; Hayashi and Fujimoto, 2010). These lipids play a crucial role in recruiting Sig-1R to the MAM (Hayashi and Fujimoto, 2010), thus enabling Sig-1R to form functional protein complexes with MAM proteins. Whether Sig-1R is involved in ER lipid synthesis is, however, just beginning to be explored. Nonetheless, it has been shown that Sig-1R knockdown suppresses glucosylceramide synthesis but upregulates HMG-CoA reductase by suppressing ER-associated degradation (Hayashi et al., 2012). Further studies are undoubtedly needed to understand the precise molecular role of Sig-1R in regulating lipid synthesis.

## Conclusion

Sig-1R unique cell biological characteristics are as follows: (1) it is an ER membrane protein which shares no homology with any other mammalian protein and forms homo-oligomers; (2) it is enriched in a specialized ER subdomain which physically contacts the mitochondria (i.e., the MAM); (3) it is activated when cells face ER or oxidative stress and ameliorates this stress, thereby promoting cellular survival; and (4) Sig-1R oligomerization, which is regulated by ligand binding, is a key factor in its activation and association with BiP. Sig-1R’s subcellular localization gives this small molecule the ability to regulate communication among organelles. Its unique mode of activation, which is triggered under ER stress or ROS accumulation, could be exploited to treat human diseases, especially those caused by misfolding proteins accumulating in the nervous system (i.e., conformational diseases such as Alzheimer’s disease, amyotrophic lateral sclerosis, and Parkinson’s disease).

## Author Contributions

TH has carried out all processes required for preparation of the manuscript.

## Conflict of Interest Statement

The author declares that the research was conducted in the absence of any commercial or financial relationships that could be construed as a potential conflict of interest.
